# SHOULDER PROBLEMS AFTER SPINAL CORD INJURY. PART ONE: SELF-ASSESSMENT BY QUESTIONNAIRES

**DOI:** 10.2340/jrm.v57.43631

**Published:** 2025-11-11

**Authors:** Ellen ANDREASSON, Jörgen SÖVERSTAD, Inka LÖFVENMARK

**Affiliations:** 1 Aleris Rehab Station, Stockholm; 2 SmärtRehab, Västmanlands Region, Västerås; 3 Karolinska Institutet, Institution for Neurobiology, Care Science and Society, Stockholm; 4 Spinalis Foundation, Stockholm, Sweden

**Keywords:** pain treatment, physical activity, prevalence, questionnaire, WUSPI

## Abstract

**Objectives:**

To describe the prevalence of -shoulder pain and associations with patient characteristics and activities of daily living in individuals with spinal cord injury (SCI) and to identify pain--relieving strategies. This paper presents questionnaire results from a larger study that also includes clinical and ultrasound assessments.

**Design:**

Cross-sectional study.

**Participants:**

Individuals with SCI for at least 1 year.

**Methods:**

A questionnaire including patient characteristics, shoulder-loading activities, and pain prevalence was distributed. Those with shoulder pain were asked additional pain-related questions.

**Results:**

One hundred participants were included (mean age=54 years, 79 male, time since SCI=13.8 years, wheelchair users=56). Shoulder pain prevalence was 34% during the last week, with higher rates after tetraplegia AIS A-D. Mode of ambulation did not affect pain ratings; however, wheelchair users experienced more bilateral pain. Pharmacological use specific for shoulder pain was low; non-pharmacological treatments included exercise, massage, and TENS.

**Conclusions:**

This study confirms a high prevalence of shoulder pain in persons with chronic SCI, with small differences across subgroups. This suggests that shoulder attention is equally important among individuals with incomplete SCI and ambulatory individuals as it is for wheelchair users. The majority with shoulder pain experienced onset of pain over 1 year ago.

## LAY ABSTRACT

This study on shoulder pain after spinal cord injury took place in Stockholm. A questionnaire was distributed including type of and time since injury, shoulder loading, shoulder pain, and pain specifics. The results showed that shoulder pain remains a substantial problem within this group (self-reported shoulder pain in the last week=34%), and was evenly distributed regardless of sex, age, and time since injury. Among those with tetraplegia, complete or incomplete, the reports were 42–44%. Wheelchair users experienced pain in both shoulders more frequently compared with ambulatory participants. Pharmacological treatment was low; however, many took medication for other kinds of pain, such as neuropathic. Training, TENS, and massage were used for pain management. This study shows that individuals with all types of SCI, including ambulators, need to take preventive measures to maintain shoulder health and need follow-up by qualified staff. Clinical and ultrasound assessments were also done as part of a larger study and will be forthcoming shortly.

Shoulder pain is a well-known common secondary condition among individuals with spinal cord injury (SCI). Reports of estimated prevalence vary; however, persons with SCI have a substantially higher prevalence of shoulder pain compared with the general population: 30–73% (1–4) vs 7–26% (5, 6). This higher prevalence is often explained by increased weightbearing for the shoulder tissues, with tasks such as wheelchair propulsion and ambulating with assistive devices (7, 8). Tetraplegia from a cervical SCI also increases vulnerability to shoulder pain due to the decreased motor and/or sensory function around the shoulder girdle (8, 9). Other risk factors that are often mentioned include duration and completeness of SCI, older age, female sex, higher body mass index (BMI), and use of a manual wheelchair (1).

Clinical examination is the most common method for diagnosis of shoulder pain/problems followed by diagnostic imaging, with MRI and ultrasound being the most common techniques (10). For clinical examination there is little agreement on what procedures and tests should be used, but the Wheelchair User's Shoulder Pain Index (WUSPI), range of motion, and strength tests are often included. A recent review article found substantial heterogeneity regarding clinical examination, with some not reporting the entire examination process and others not indicating what factors determined the test findings as positive or negative (10). Ultrasound is becoming more available and commonly used and can be a valuable complement to the physical examination (10). It can also be used for dynamic -assessments.

Treatment options for shoulder pain include pharmacological, physiological, psychological, and surgical interventions. In the SCI population, analgesics are commonly used for neuropathic and/or musculoskeletal pain with drugs such as gabapentin, amitriptyline, NSAIDs, paracetamol, and opioids (11) as well as cortisone injections (12). Non-pharmacological treatments such as exercise, acupuncture, and massage are commonly used (11) and positive effects have been reported (13–15). Surgical options, including acromioclavicular joint surgery, subacromial decompression, tendon repair, or glenohumeral joint arthroplasty can be considered when conservative treatment fails (16).

Over the past years, the heterogenic population of individuals with SCI has changed characteristics based on clinical experiences, and this has been verified from the Swedish register for rehabilitation medicine (17). Incomplete injuries, tetraplegia, and higher age are more common (17); the degree to which shoulder problems/pain affect this group is not well charted. The effect of pain-prevention strategies that have been implemented to decrease shoulder problems is also often not fully evaluated. There are gaps in knowledge because many studies are small and from different contexts (10).

In the Stockholm region, a prevalence study of shoulder pain among wheelchair users with thoracic SCI (*n*=88) was conducted in 2008 (4) and showed a 40% prevalence of current shoulder pain. The authors pointed out the need to develop prevention programmes and to strengthen the existing patient education. They also emphasized prioritizing early diagnosis, prompt treatment of shoulder pain, and advised more frequent use of assistive devices. Since then, the patient characteristics have altered, and the average patient is older, and has more incomplete injuries and tetraplegia. Therefore, this study covers the whole heterogenic group of individuals with SCI and maps the prevalence of shoulder pain and specific pathologies. It is hoped that the analysis of subgroups can contribute to differentiating follow-up interventions to be more person-centred when it comes to risk factors and identify quantitative, easily used clinical measures. This could also be the basis of a quality register that could be followed over time. We did not find any similar study that in the data collection and analysis included surveys, measuring instruments, clinical assessments, and ultrasound assessments on shoulders bilaterally in order to provide an overall picture of the complexity regarding shoulder problems after SCI.

This is the first of 3 parts of the study “Shoulder problems after spinal cord injury”, including self-reported results regarding shoulder pain. Part II will include results from the clinical assessment and ultrasound investigations, and Part III includes results from measurements of flexibility, muscle strength, scapula testing, and positioning.

The aims of this study, Part I, were to describe the prevalence of shoulder pain and its associations with demographic and clinical characteristics in individuals with all types of SCI in the Stockholm region. Additionally, the intention was to explore whether shoulder pain correlates with daily activity levels or use of technical aids, and pain-relieving strategies.

## MATERIAL AND METHODS

### Setting

In Sweden there are approximately 300 new cases of acquired SCI annually and about 80 individuals underwent rehabilitation in Stockholm in 2018 (12). All individuals with SCI in the Stockholm region (approximately 1,400 persons) are registered at the Spinalis outpatient clinic (Aleris Rehab Station) and are routinely scheduled for multidisciplinary long-term follow-up, aiming to maintain health, oversee technical aids and medications, and detect, prevent, and treat secondary complications.

### Study design and population

This study included individuals with SCI in the Stockholm region who attended routine follow-ups at the Spinalis outpatient clinic between May 2022 and September 2023. Inclusion criteria were acquired traumatic or non-traumatic SCI at least 1 year ago; age 18–75 (to prioritize SCI-related problems instead of age-related problems); and the ability to understand Swedish. Exclusion criteria included (in ranked order) first visit to the clinic (unclear regarding SCI and registration at the clinic); requiring interpreter; severe psychological or cognitive impairment; or other unspecified reasons such as being transported on a stretcher. Notably, shoulder pain was not a criterion for inclusion or exclusion.

### Methods

During routine follow-ups (scheduled by a person not involved in the project), eligible individuals received verbal and written study information and were consecutively included in the study. Those who declined participation were asked for consent to use background data from their patient files (sex, age, level and completeness of SCI, and presence of shoulder pain) for dropout analysis. The study protocol included a questionnaire, clinical assessment, and ultrasound assessment of the shoulder joints (Table I).

**Table I:** Overview of data collection variables for the 3 parts of the study “Shoulder problems after spinal cord injury”. This paper addresses data based on the questionnaire (part I)

Assessments	Data collection	Study part
Baseline data	• Age, sex • Neurological level of injury and completeness • American Spinal Injury Association (ASIA) motor score • Presence of pain post-SCI	I–III
Questionnaire for all participants (*n*=100 participants)	• Demographic characteristics (living circumstances, employment rate, physical activity) • Clinical characteristics (time since injury, weight, mode of ambulation, transfers, driving) • Medication for other pain than shoulder pain	I
Additional questionnaire for those who reported shoulder pain post SCI (*n*=54 participants)	• History of shoulder pain, onset, cause o sought healthcare for shoulder pain – where/from whom o previous assessments, treatment and/or medication o previous shoulder trauma • ISCoS pain data set (page 1: pain ratings/interference of pain) • Wheelchair User's Shoulder Pain Index (WUSPI) • Shoulder Rating Questionnaire (SRQ-S) • Pain drawing	I
Clinical assessment when sitting (*n*=200 shoulders)	• Active range of motion (AROM) • Clinically relevant shoulder tests (Jobes test, Hawkins test, Speeds test, painful arch, crossover test, wing scapula test, and side differences in isometric strength. Foramen compression and palpation of shoulder structure • Neuromuscular tests for impingement	II
Ultrasound assessment when sitting (*n*=200 shoulders)	• Documentation of tendons (subscapularis, supraspinatus, infraspinatus, biceps) with thickening, calcification, and partial or full-thickness tears • Cortical irregularities (osteoarthrosis) of the AC and GH joints or increased amount of fluid in the joint. Paralabral cysts • Abnormalities of the subacromial/subdeltoid bursa: bursitis (increased fluid) or fibrotic (thickened).	II
Measurements when sitting and supine (*n*=200 shoulders)	• Angles of the medial border of the scapula (digital goniometer) • Distances between the acromion in normal and retracted position (measuring tape) • Passive range of motion (PROM) (digital goniometer) • Isometric muscle strength (digital myometer)	III
Positioning (*n*=100 participants)	• Photographs for assessment of the sitting posture and symmetry • Posture and Postural Ability Scale (PPAS) was used to document posture when sitting	III

Initially the assessment protocol was tested on 1 person with SCI who was not included in the study followed by some modifications of the protocol. A pilot study with 5 participants followed, leading to minor adjustments of the protocol after which the participants were included in the study.

### Variables and data collection

Table I presents the data collected in the full study and illustrates the divisions into a 3-part study. The questionnaire was partly based on the International Spinal Cord Society (ISCoS) data sets (Socio-demographic, and Activity and participation data) (18) and the Spinal Cord Independence Measure (SCIM) for functional level (19) and was distributed to all participants.

For those with shoulder pain post-SCI, an additional questionnaire was distributed with specific questions on shoulder pain. Finally, the following pain rating instruments were distributed:

- ISCoS pain data set (18) with Numeric Ratings Scale (NRS) (0–10 represents no pain to worst possible pain), and how the pain affects activities in daily life (ADL), mood, and sleep were rated for all participants with pain in the last week.
- Wheelchair User's Shoulder Pain Index (WUSPI) (20, 21) was distributed to wheelchair users. This validated self-report questionnaire assesses shoulder pain during 15 activities over the past week using a Visual Analogue Scale (VAS) (0–100). WUSPI is a validated and commonly used instrument for individuals with SCI and shoulder pain (22). Performance-corrected WUSPI was calculated to adjust for missing data or tasks that the patient did not perform and therefore did not answer.
- Shoulder Rating Questionnaire-Swedish (SRQ-S) (23) was distributed to ambulatory participants with shoulder pain. This instrument is constructed for the general population and targets shoulder pain or limitations in different activities during the last month. The full instrument includes 21 questions; however, 4 questions were included in the questionnaire for all participants with pain.

Time points for shoulder pain ratings were at any time after SCI, last month, last week, and current pain. The last month time point is limited to SRQ-S ratings, and current pain (on the day of assessment) were considered to fall under pain last week and were not analysed further. Therefore, the primary time points that were analysed and presented are shoulder pain at any time after SCI and pain during the last week.

### Statistics

All data were coded and transferred to the IBM SPSS statistical programme for analysis (Version 28.0.0.0; IBM Corp, Armonk, NY, USA). Age was grouped into 18–30, 31–45, 46–60, and 61–75 (24). Level and completeness of SCI were sub-grouped based on the International Standards for Neurological Classification of SCI (ISNCSCI) and the American Spinal Injury Association Impairment Scale (AIS) (25) as tetraplegia C1–4 and C5–8 AIS A-C, paraplegia AIS A-C, and all AIS D (23), partly divided in to tetra-/paraplegia AIS D. Participants were classified as wheelchair user, ambulatory with assistive devise, or ambulatory without assistive device depending on what mode of ambulation they used 75% of the time. Time since SCI was reported in years and time since onset of shoulder pain in months. Descriptive statistics are presented as absolute numbers and proportions for categorical variables, and for continuous variables as mean and standard deviation (SD). Inferential statistics were used for group comparisons, a χ^2^ test for categorial variables, and a *t*-test for continuous variables. Non-parametric tests, Mann–Whitney *U* test, Kruskal–Wallis test, and Pearson χ^2^ test were used due to small groups. *P*-value was set to ≤0.05. Dropout were conducted analyses on eligible individuals who declined participation.

## RESULTS

### Descriptives

**Fig. 1:**
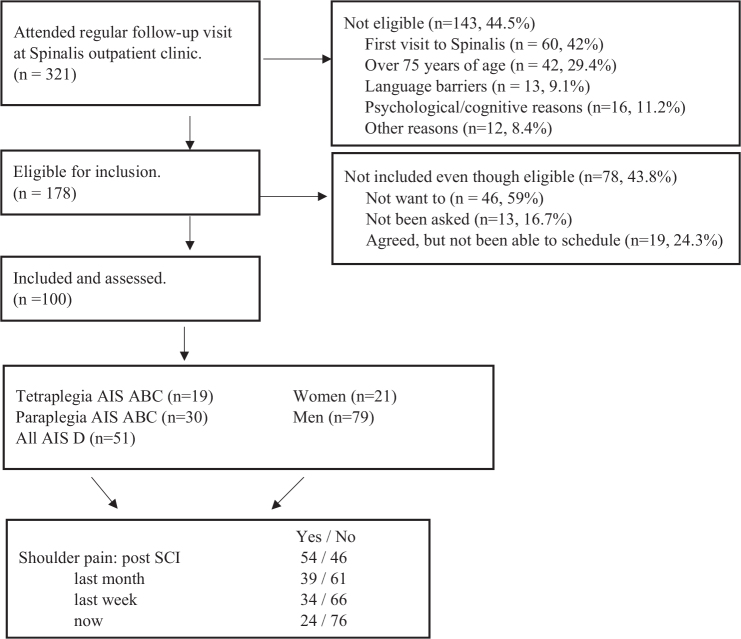
Flowchart of the inclusion process.

Over 300 individuals were attending structured follow-up during the inclusion period and of those, 178 were eligible for inclusion. One hundred participants were included in the study (Fig. 1), with a mean age of 54 years (range 19–75) (Table II). Time since injury was in mean 13.8 years, ranging between 1 and 59 years with significantly longer duration especially for those with tetraplegia C1-4 AIS A-C compared with other types of SCI (*p*=0.028) and for wheelchair users compared with ambulatory participants (*p*=0.013) (Table III).

**Table II:** Sociodemographic and clinical characteristics of participants with or without shoulder pain after SCI at all and during the last week

Variables	Total (*n*=100)	Participants with shoulder pain after SCI (*n*=54)	Participants without shoulder pain after SCI (*n*=46)	*p*-values Pain or not pain post-SCI	Participants with shoulder pain last week (*n*=34)	Participants without shoulder pain last week (*n*=66)	*p*-values Pain or no pain last week
Sex, *n* (%)				*p*=0.249^b^			***p*=0.032^b^**
Female	21	9 (42.9)	12 (57.1)		3 (14.3)	18 (85.7)	
Male	79	45 (57.0)	34 (43.0)		31 (39.2)	48 (60.8)	
Age, years, mean (SD)	54.0 (14.8)	53.4 (14.0)	54.7 (15.8)	*p*=0.695^a^	52.3 (14.7)	54.9 (14.8)	*p*=0.405^a^
16–30 years, *n* (%)	6	2 (33.3)	4 (66.7)		2 (33.3)	4 (66.7)	
31–45 years, *n* (%)	19	13 (68.4)	6 (31.6)		8 (42.1)	11 (57.9)	
46–60 years, *n* (%)	40	21 (52.5)	19 (47.5)		14 (35.0)	26 (65.0)	
61–75 years, *n* (%)	35	18 (51.4)	17 (48.6)		8 (22.9)	27 (77.1)	
Time since injury (years), mean (SD)	13.8 (13.7)	13.5 (13.4)	14.3 (14.1)	*p*=0.766^a^	11.0 (10.5)	15.3 (14.9)	*p*=0.135^a^
Level and completeness of injury, *n* (%)				*p*=0.762^b^			*p*=0.854^b^
Tetraplegia C1–4 AIS A–C	7	5 (71.4)	2 (28.6)		3 (42.9)	4 (57.1)	
Tetraplegia C5–8 AIS A–C	12	7 (58.3)	5 (41.7)		5 (41.7)	7 (58.3)	
Paraplegia AIS A–C	30	15 (50.0)	15 (50.0)		9 (30.0)	21 (70.0)	
All AIS D	51	27 (52.9)	24 (47.1)		17 (33.3)	34 (66.7)	
Tetraplegia AIS D	25	15 (60.0)	10 (40.0)		11 (44.0)	14 (56.0)	
Paraplegia AIS D	26	12 (46.2)	14 (53.8)		6 (23.1)	20 (76.9)	
Motor score, mean (SD)	67.3 (26.9)	65.4 (27.7)	69.6 (26.0)	*p*=0.434^a^	63.2 (28.0)	69.5 (26.2)	*p*=0.268^a^
Aetiology, *n* (%)				*p*=0.240^b^			*p*=0.387^b^
Traumatic SCI	71	41 (57.7)	30 (42.3)		26 (36.6)	45 (63.4)	
Non-traumatic SCI	29	13 (44.8)	16 (55.2)		8 (27.6)	21 (72.4)	
Body mass index, mean (SD)	25.7 (5.2)	25.5 (5.4)	25.8 (5.0)	*p*=0.744^a^	25.8 (5.6)	25.6 (5.0)	*p*=0.864^a^
Underweight*, *n* (%)	2	1 (50.0)	1 (50.0)		1 (50.0)	1 (50.0)	
Normal weight*, *n* (%)	43	25 (58.1)	18 (41.9)		16 (37.2)	27 (62.8)	
Overweight*, *n* (%)	33	17 (51.5)	16 (48.5)		11 (33.3)	22 (66.7)	
Obesity*, *n* (%)	15	7 (46.7)	8 (53.3)		4 (26.7)	11 (73.3)	
Severe/Very severe obesity*	7	4 (57.1)	3 (42.9)		2 (28.6)	5 (71.4)	
Physically active: Yes/No, (*n*)	80/20	44/10	36/10	*p*=0.688^b^	26/8	54/12	*p*=0.527^b^
Living circumstances^c^, *n* (%)				*p*=0.704^b^			*p*=0.946^b^
Living together**	53	28 (52.8)	25 (47.2)		17 (32.1)	36 (67.9)	
Living alone***	46	26 (56.5)	20 (43.5)		17 (37.0)	29 (63.0)	
Other	1	1 (100)	0 (0)		0 (0)	1 (100)	
Number in household, mean (SD)		1.9 (1.0)	2.2 (1.2)	*p*=0.314^a^	1.9 (1.0)	2.1 (1.1)	*p*=0.570^a^
1–2	80	44 (55.0)	36 (45.0)	*p*=0.411^b^	27 (33.7)	53 (66.3)	*p*=0.266^b^
3–4	15	9 (60.0)	6 (40.0)		7 (46.7)	8 (53.3)	
5–7	5	1 (20.0)	4 (80.0)		0 (0)	5 (100)	
Employment rate, *n* (%)^c^				*p*=0.168^b^			*p*=0.124^b^
Employed/student	48	24 (50.0)	24 (50.0)		16 (33.3)	32 (66.7)	
Unemployed	5	2 (40.0)	3 (60.0)		2 (40.0)	3 (60.0)	
Retired	31	16 (51.6)	15 (48.4)		7 (22.6)	24 (77.4)	
On sick leave	14	10 (71.4)	4 (28.6)		7 (50.0)	7 (50.0)	
Other	2	2 (100)	0 (0)		2 (100.0)	0	
Wheelchair/ambulatory, *n*	56/44	32/22	24/22	*p*=0.477^b^	20/14	36/30	*p*=0.683^b^
Wheelchair, *n*							
Manual wheelchair	37	20	17		12	25	
Electric wheelchair	3	2	1		2	1	
Both manual and electric	11	8	3		4	7	
Assisted manual	4	2	2		2	2	
All of the above	4	3	1		2	2	
Walking with shoulder loading aids****	30	15	15		10	20	
Walking without assistive device	14	7	7		4	10	
Drive car Yes/No, *n*	62/38	33/21	29/17	*p*=0.843^b^	21/13	41/25	*p*=0.972^b^

Significant differences are shown in bold.

* Adapted BMI to paraplegia and tetraplegia.

** Including married and living together.

*** Including single, living apart, separated, widow/widower.

**** Walking aids include cane/crutch (*n*=26), walker (*n*=8), poles (*n*=10). Multiple aids used by 9. Orthotics used: ankle–foot orthosis (*n*=4), knee support (*n*=1), or full-leg orthosis (*n*=1).

a *t*-test,

b χ^2^ test.

c *p*-value calculated separately, presented grouped.

AIS: American Spinal Injury Association Impairment Scale; SD: standard deviation; SCI: spinal cord injury.

**Table III:** Pain ratings during rest and activities at measurement points last week and last month

Variables	Total group *n*=100	Tetraplegia C1–4 AIS A-C *n*=7	Tetraplegia C5–8 AIS A-C *n*=12	Paraplegia AIS A–C *n*=30	AIS D *n*=51	W/C users *n*=56	Ambulatory with AD *n*=30	Ambulatory without AD *n*=14
Pain post SCI, *n* (%)	54 (54)	5 (71.4)	7 (58.3)	15 (50.0)	27 (52.9)	32 (57.1)	15 (50.0)	7 (50.0)
Time since SCI, years, mean (SD)*	13.8 (13.7)	**24.6 (19.9)**	**15.4 (13.9)**	**16.6 (12.7)**	**10.4 (12.3)**	**17.4 (14.9)**	**9.5 (10.9)**	**8.8 (9.4)**
Onset of pain, months, mean (SD)*	76.5 (84.9)	144.0 (69.5)	64.7 (102.7)	72.1 (82.1)	69.0 (82.8)	78.0 (84.1)	65.6 (76.8)	91.4 (111.8)
NRS ratings pain last week** Mean (SD)		*n*=3	*n*=5	*n*=7–9	*n*=17	*n*=19	*n*=10	*n*=4
Affect daily activities (*n*=32)	4.0 (2.6)	3.7 (2.1)	4.4 (4.4)	3.9 (2.8)	4.1 (2.1)	3.9 (2.9)	4.1 (2.6)	4.2 (1.3)
Affect mood (*n*=33)	3.7 (3.0)	3.7 (3.8)	4.6 (4.2)	3.4 (3.5)	3.5 (2.5)	3.7 (3.4)	3.3 (3.0)	4.2 (1.7)
Affect sleep (*n*=34)	3.4 (2.6)	2.3 (1.5)	2.8 (3.1)	4.2 (3.0)	3.3 (2.4)	3.4 (2.8)	3.5 (2.6)	3.0 (2.2)
Average pain intensity (*n*=32)	4.5 (2.5)	3.7 (2.1)	5.6 (3.4)	3.7 (2.8)	4.6 (2.1)	4.1 (2.7)	4.4 (2.2)	6.2 (1.3)
Pain last month*** *n*=39, *n* (%)		*n*=2	*n*=5	*n*=11	*n*=21	*n*=21	*n*=13	*n*=5
How would you rate your pain at rest?								
None	6 (15.4)	0	1	2	3	5	1	0
Weak/medium	23 (59.0)	2	2	5	14	10	8	5
Severe/very severe	10 (25.6)	0	2	4	4	6	4	0
How would you rate your pain during activities?								
None	0 (0)	0	1	0	0	0	0	0
Weak/medium	28 (71.8)	2	2	7	16	15	9	4
Severe/very severe	11 (28.2)	0	2	4	5	6	4	6
Considering your shoulder, how do you manage to perform everyday life activities?								
No limitations	13 (33.3)	0	1	4	8	7	5	1
Some/moderately limited	23 (59.0)	2	3	7	11	13	6	4
Severely/very severely limited	3 (7.7)	0	1	0	2	1	2	0
Considering your shoulder, how do you manage to perform leisure time and sports activities?								
No limitations	7 (18.0)	0	2	1	4	4	3	0
Some/moderately limited	22 (56.4)	1	2	6	13	10	8	4
Severely/very severely limited	10 (25.6)	1	21	4	4	7	2	1

Significant differences are shown in bold.

* Time since injury/type of SCI *p*=0.028, time since injury/mood of ambulation *p*=0.013. Onset of pain/type of SCI *p*=0.101, onset of pain/method of ambulation *p*=0.910.

** Derived from International Spinal Cord Society pain data set. No significant differences between type of SCI or method of ambulation.

*** Derived from Shoulder Rating Questionnaire-Swedish. No significant differences between any of the 4 SRQ-S ratings and type of SCI or method of ambulation.

AD: assistive device; AIS: American spinal Injury Association Impairment Scale; NRS: Numeric Rating Scale; SD: standard deviation; W/C: wheelchair.

Background data for dropouts were collected from 51 individuals (out of 78) who were eligible but did not participate. The mean age was 53.9 years (range 26–75); 32 were men. Sixteen individuals had a cervical SCI and 31 had an AIS D SCI. Thirteen reported shoulder pain, 26 denied pain (missing data=13). Reasons for not participating are presented in Fig. 1.

### Pain ratings

Fifty-four (54%) of the participants had experienced shoulder pain at any time after SCI. Of those with tetraplegia AIS A–C and D approximately 60% reported shoulder pain. Pain during the last week was reported by 34 participants and among the group of tetraplegia AIS A–C and D rates were over 40% (see Table II). There were no significant differences between those with or without pain after SCI regarding sex, age, time since SCI, level or completeness of injury, aetiology, BMI, reported physical activity, or living circumstances (Table II), except that wheelchair users had more bilateral pain compared with ambulatory participants (*p*=0.039). There were also no differences between those with or without shoulder pain in the last week, except a higher rate of shoulder pain among men as compared with women (Table II).

The mean time for onset of pain was 76.5 months, median 24, range 1 month–25 years, with 4 having an onset of less than 3 months. Participants with C1–4 AIS A–C injuries seemed to have had longer time since onset of pain compared with the other groups; however, analysis showed no significance (*p*=0.101). There were no differences regarding onset of pain between wheelchair users and those who were ambulatory (*p*=0.910) (see Table III).

**Fig. 2:**
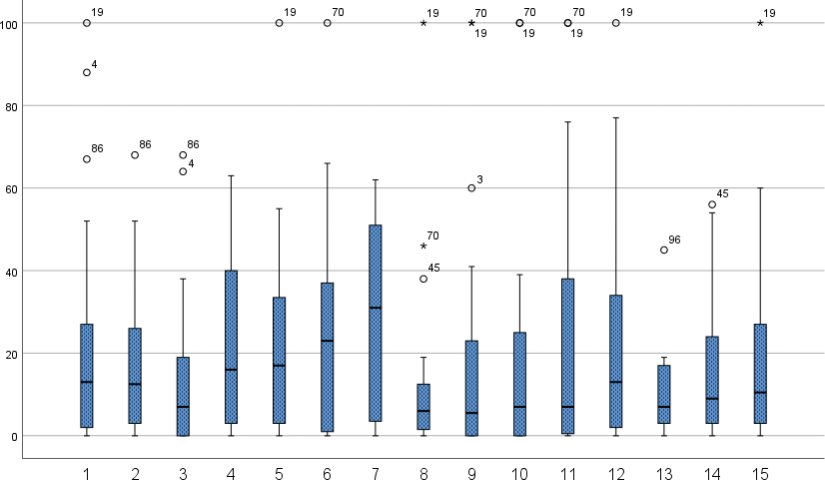
Boxplot for individual Wheelchair User's Shoulder Pain Index (WUSPI) scores for all wheelchair users who had shoulder pain at any time post spinal cord injury (*n*=27). Boxplot shows the median, interquartile range, and total population with outliers (1: transferring from bed to wheelchair; 2: transferring from a wheelchair to a car; 3: transferring from a wheelchair to tub or shower; 4: loading wheelchair into a car; 5: pushing your wheelchair for ≥10 min; 6: pushing up inclines; 7: getting down objects from overhead shelf; 8: putting on trousers; 9: putting on a T-shirt or pullover; 10: putting on a button-down shirt; 11: washing your back; 12: usual daily activities at work/school; 13: driving; 14: performing household chores; 15: sleeping).

**Fig. 3:**
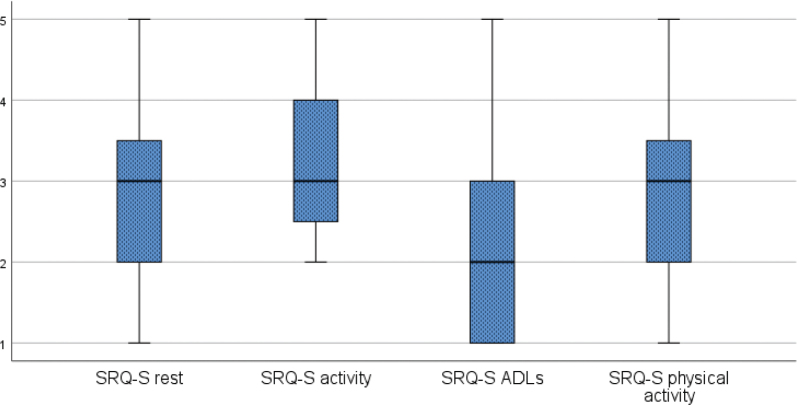
Boxplot for individual Shoulder Rating Questionnaire-Swedish (SRQ-S) ratings for all participants with pain the last month (*n*=39). Boxplot shows the median, interquartile range, and total group. Pain is rated according to a 5-step scale from “no pain (1)–very severe pain (5)” or “no limitations (1)–very severely limited (5)”. (1. Rest: how would you describe your shoulder pain at rest?, 2. Activity: how intense was your pain during activities?, 3. ADLs: considering your shoulder, how do you handle daily activities such as dressing, grooming, driving, household chores etc.?, and 4. Physical activity: considering your shoulder, how do you manage leisure time and sports activities?).

Of the 34 participants reporting pain in the last week, 18 reported pain 7 days/week and pain intensity was rated as a mean 4.5/10 on NRS (see Table III). Ratings on how pain affected their ADL, mood, and sleep last week are presented in Table III and showed no significant differences between groups with regard to injury or being a wheelchair user or ambulatory. WUSPI ratings were done by 27 wheelchair users (missing data=5) with shoulder pain. The pain intensities were highest when lifting objects above the head (#7) (mean 32.2), followed by propelling on ramps or slopes (#6) (mean 27.1) (Fig. 2). SRQ-S ratings were conducted by the 39 participants who experienced pain in the past month and showed that “performing activities” had the highest minimum value and was skewed towards “very severe pain” (Fig. 3).

Shoulder trauma was reported by 26% (14/54) of participants with pain after SCI. Among those with shoulder trauma before SCI (*n*=7), 2 participants reported onset of shoulder pain before the SCI injury and 5 after. Similarly, of those with shoulder trauma post-SCI (*n*=7), 1 participant reported the onset of shoulder pain prior to the SCI and 6 developed it post-SCI. -Trauma details (e.g. clavicular/scapular fractures, bicycle accidents) were reported but not analysed statistically due to the small sample.

Finally, 80 participants reported being physically active, of whom 44% reported shoulder pain, i.e. no association could be detected. The most common activities were walking/propulsion, cycling, and gym-training.

### Pain treatments

Analgesics were used regularly by 40 participants (40%), of whom 27 used analgesics that are recommended for neuropathic pain (gabapentin, pregabalin, duloxetine, amitriptyline). Opioids were used by 13 participants, alone or in combination with the above-mentioned drugs. Paracetamol and NSAIDs, normally used for treatment of nociceptive musculoskeletal pain, were regularly used by 15%.

Of the 54 participants who reported shoulder pain after SCI, 28 had sought healthcare for their pain, primarily by physiotherapists and/or physicians, mainly at the Spinalis outpatient clinic (*n*=25). Nine participants confirmed that they used pharmacological treatment for or against shoulder pain, including opioids (*n*=3), paracetamol (*n*=2), injection of corticosteroids (*n*=6), and/or NSAIDs per os/gel (*n*=2). Non-pharmacological pain relief strategies were used by 25%, including exercise (*n*=9), massage (*n*=11), TENS (*n*=10), or stretching (*n*=8). Ten participants had tried 2–3 different alternatives and 8 used more than 4 different non-pharmacological treatment strategies, therefore it was not possible to analyse the effectiveness of each. Two participants had undergone shoulder surgery: 1 Bankart repair after subluxation and 1 arthroscopic capsular release.

## DISCUSSION

The main findings of this study were that over half of the participants had experienced shoulder pain after their SCI and one-third reported shoulder pain during the last week, with non-statistically significant small differences between subgroups (age, sex, type of SCI). The majority had experienced pain for over 1 year and wheelchair users had more bilateral shoulder pain compared with ambulatory participants.

Just over half of the participants, 54%, reported shoulder pain at some point after their SCI, which is well in line with previous reports (1). Pain during the last week was reported by 34%, which is lower compared with previous studies (2, 4) but comparable with a recently published study by Bossuyt et al. (26). This somewhat lower rate might be the result of improved prevention strategies, improved technical aids, especially lightweight wheelchairs and motorized help-wheels are more widely available in the Stockholm area. However, it is hard to determine the precise reason for this small decrease since those variables are not isolated and changes were not part of this study.

We found no to few associations between shoulder pain and demographic characteristics. However, men did report more pain in the last week compared with women. This difference is contrary to existing literature showing higher pain prevalence among individuals with SCI in women (3, 26). This discrepancy was only shown during one measurement point (last week) and might be due to the fact that the subgroup of women was small and statistical analysis in this regard should be interpreted with caution.

Contrary to findings from previous studies (1, 27), there was no association regarding age and self-reported shoulder pain. The mean age of the participants was relatively high, which could increase the risk of shoulder pain due to general degenerative changes associated with ageing.

Shoulder pain was also evenly distributed over subgroups of level and severity of injury, which was surprising. As presented in Table II, we did undertake a detailed analysis but most comparisons showed non-significant differences. We still choose to include all data in the table to be transparent. Previous studies have pointed out an increased vulnerability to develop shoulder dysfunction among persons with complete tetraplegia (8, 9). In this study we saw a tendency for more pain among participants with tetraplegia, complete or incomplete, and especially those with C1–4 injuries, but the differences were not statistically significant. A recently published large study showed a 32% prevalence of self-reported shoulder pain iin the last week among 2,772 individuals (26), which is in line with our study. That study’s subgroups also showed the same pattern as our study, with a higher prevalence of shoulder pain among those with incomplete tetraplegia compared with incomplete paraplegia even though the difference was larger in our study, at 44% vs 23%.

Increased weightbearing on the shoulders, especially through wheelchair use but also ambulation with assistive devices, is often reported as a big risk factor for shoulder pain (27). This was not shown in our study. However, wheelchair users presented more frequently with bilateral shoulder pain compared with ambulating participants. This may be due to several factors: (*i*) wheelchair users had lived with their SCI for a longer duration which might have impacted shoulder overuse, (*ii*) when unilateral should pain arises, wheelchair dependency will lead to a heavy burden on the other shoulder, and (*iii*) wheelchair users are simultaneously loading bilateral shoulders in a different way from ambulators. We have not been able to find any previous studies on this topic.

Approximately half of the participants had AIS D SCI with mild to moderate neurological impairment. However, only 14 participants were ambulatory without assistive devices, indicating a substantial presence of neurological deficits among the participants. Half of the participants with AIS D injury had tetraplegia, and they seem to have a similar rate of shoulder pain to those with AIS A–C. This finding strengthens the need for prevention strategies and follow-up for persons with more incomplete SCI similar to those for individuals who are wheelchair users.

Weight-bearing tasks such as transfers and physical activity were included in the questionnaire to detect whether overuse was related to pain. We could not detect any associations between shoulder pain and level of activity; however, the results were inconclusive. Answers have been hard to interpret regarding the amount of transfers/day and the majority (80%) claimed to be physically active, even though the level of activity was uncertain. Considering the lack of correlation between being physically active and experiencing shoulder pain, we cannot distinguish whether physical activity in this case was beneficial or detrimental.

When comparing the results of our study with those of Alm et al. (4) conducted at the same clinic in 2008 and which included 88 full-time wheelchair users with motor complete thoracic SCI, we focused on the subgroup of full-time wheelchair users with paraplegia (AIS A–C, *n*=29). The reports of shoulder pain at all 4 points of measurement seem to be slightly lower compared with the 2008 study, with 67% of participants reporting pain after SCI vs 52% in the current study, and 41% vs 31% reporting pain in the last week. This is a promising result, even though shoulder pain remains a substantial problem area for this population. However, our sample was smaller (*n*=29), included participants with AIS C injuries (*n*=3), and statistical comparisons were not conducted. Alm et al. highlighted the need for prevention programmes, patient education, prompt diagnosis, prioritizing treatment of shoulder pain, and the use of more assistive devices for transfers and wheelchair propulsion. Since then, preventive strategies have been strengthened in rehabilitative care, including patient education on shoulder function, preventive strength training, increased use of transfer boards, “wheelchair school” focusing on propulsion techniques, and accurate wheelchair adjustments. Questions regarding prevention and rehabilitation strategies were not addressed specifically in this study questionnaire but these measures are included for all patients undergoing rehabilitation in Stockholm. Further, advancements in assistive technology, such as lightweight wheelchairs and more widespread provision of motorized wheels to assist in longer distance propulsion, have become more common.

Nine participants confirmed that they used pharmacological treatment for shoulder pain, which was surprisingly few, especially regarding the use of NSAIDs. One reason for this might be the relatively common use of analgesics for reasons other than shoulder pain, such as neuropathic pain. However, non-pharmacology treatment was more commonly used including exercise, massage, and acupuncture. Exercise has shown good benefits regarding shoulder pain (28); however, long-term effects were not evaluated. Considering that 80% of participants reported engaging in physical activities such as walking/propulsion, gym training, cycling/arm cycling, and pool exercises, the report of exercise as a treatment may have been underreported, i.e., that the participants did not see the exercise as a form of treatment, perhaps more as a preventative measure.

Individuals with SCI constitute a heterogeneous group with varied loading profiles on their shoulders and various functional conditions regarding, for example, weakness and immobilization (29). There are also different time points to assess shoulder pain, from the onset of shoulder pain after SCI to current pain and a variation of outcome measures such as NRS, WUSPI, and SRQ-S (10). With that consideration we believe it is important to follow shoulder pain on an individual level, with standardized data quality registers and clinical differentiation in collected data dependent on SCI subgroup such as wheelchair user or ambulatory. When comparing results from different studies we believe that it could facilitate the follow-up process and be beneficial when conducting longitudinal studies to have a standardized time point to assess shoulder pain, such as “pain in the last week”, which we found to be a relevant outcome measure.

The clinical implications of the results from this study pinpoint that the need for prevention strategies and a follow-up structure for shoulder pain is equally important after all types of SCI, even for ambulatory individuals. Due to the evenly distributed pain prevalence over the subgroups, previously established risk factors might need to be revised. Finally, protocols for shoulder assessments over time need to be standardized and registered to facilitate long term follow-up.

### Strenghts and limitations

A strength of this study was that the survey was filled out at the clinic resulting in minimal missing data. Also, that the inclusion of participants with various types of acquired SCI reflected the diversity in a specialized SCI unit. While many studies have primarily focused on individuals with complete paraplegia, this study provides a much warranted update. However, this can also be a limitation, as heterogenicity can cause small subgroups. In this study, the high rate of participants with AIS D became a challenge with regard to other subgroups becoming small, resulting in some unreliable statistics especially for those with more extensive SCI. This high rate of AIS D, however, represents the current patient characteristics in the Stockholm region where incomplete tetraplegia has increased during the last decade (17). Despite these limitations, the results of this study can provide an updated view of shoulder problems but might not be wholly representative and transferable on the full population of patients within the Stockholm region or internationally.

### Conclusion

This study confirms a high prevalence of shoulder pain in individuals with SCI and specifically for individuals with high tetraplegia AIS A–D. It revealed no to small differences between subgroups of sex and mode of ambulation, suggesting that commonly described risk factors might have changed with the change in patient characteristics. More attention to shoulder health might also be needed for those with more incomplete injuries. Most of the participants with shoulder pain had onset of pain over 1 year ago and wheelchair users had a higher rate of bilateral shoulder pain compared with ambulatory participants. Part II and III of this study will present results from clinical and ultrasound assessments.
